# Vertical nanowire probes for intracellular signaling of living cells

**DOI:** 10.1186/1556-276X-9-56

**Published:** 2014-02-03

**Authors:** Ki-Young Lee, Ilsoo Kim, So-Eun Kim, Du-Won Jeong, Ju-Jin Kim, Hyewhon Rhim, Jae-Pyeong Ahn, Seung-Han Park, Heon-Jin Choi

**Affiliations:** 1 Department of Materials Science and Engineering, Yonsei University, Seoul 120-749, Republic of Korea; 2 Spin Convergence Research Center, Korea Institute of Science and Technology, Seoul 139-791, Republic of Korea; 3 Department of Physics, Chonbuk National University, Jeonju 561-756, Republic of Korea; 4 Center for Chemoinformatics Research Center, Korea Institute of Science and Technology, Seoul 139-791, Republic of Korea; 5 Advanced Analysis Center, Korea Institute of Science and Technology, Seoul 139-791, Republic of Korea; 6 Department of Physics, Yonsei University, Seoul 120-749, Republic of Korea

**Keywords:** Silicon nanowire, Intracellular interfacing, Living cell, Nanowire probe, Action potential

## Abstract

The single living cell action potential was measured in an intracellular mode by using a vertical nanoelectrode. For intracellular interfacing, Si nanowires were vertically grown in a controlled manner, and optimum conditions, such as diameter, length, and nanowire density, were determined by culturing cells on the nanowires. Vertical nanowire probes were then fabricated with a complimentary metal-oxide-semiconductor (CMOS) process including sequential deposition of the passivation and electrode layers on the nanowires, and a subsequent partial etching process. The fabricated nanowire probes had an approximately 60-nm diameter and were intracellular. These probes interfaced with a GH_3_ cell and measured the spontaneous action potential. It successfully measured the action potential, which rapidly reached a steady state with average peak amplitude of approximately 10 mV, duration of approximately 140 ms, and period of 0.9 Hz.

## Background

The probing of an electrical activity in extracellular and intracellular modes at a single-cell level is crucial for understanding the whole nervous system [1-5]. In this respect, neuro-physiologists have investigated a small number of cells that are grown in defined patterns, allowing for the stimulation and recording of electrical activity of individual neurons [6-9]. However, these approaches are limited in precisely probe neural activity on a single-cell level. Conventional methods of electrophysiological measurement, which use micro-size electrodes such as electrolyte-filled glass pipettes and metal wires, are useful for identifying the electrical activity of electrogenic cells with a good signal-to-noise ratio and temporal resolution [10-12]. For all these advantages, it is difficult to achieve long-term signaling, repetitive monitoring, and multi-site recording. Other alternatives, such as multi-electrode arrays and planar FET devices [13-16], also have limitations in terms of the size of the probes used for signaling cell activity without cell damage.

Meanwhile, nanomaterials can potentially be exploited to achieve ultra-high sensitivity for various label-free biosensing applications as well as in direct probing of living cell activities [17-20]. Among nanomaterials developed to date, nanowires in particular have high aspect ratios, surface areas, and very small diameters on a sub-100-nm scale. Thus, they are ideal building blocks for probing single cell activity on a submicron scale. Notably, few studies have probed electrical activity (i.e., action potential) in an extracellular mode by using horizontal nanowire transistors [7,21]. Probing the neural activity in an intracellular mode is also promising because the nanowire size is sufficiently small to provide an intracellular interface with neural cells without cell damage [22,23]. Herein, we report the interfacing of neural cells with vertical Si nanowires and the probing of neural activity in an intracellular mode on a single-cell level.

## Methods

### Synthesis of nanowires

Vertical Si nanowires were grown on Si substrates using a vapor–liquid-solid mechanism with the assistance of Au colloid particles using a low pressure chemical vapor deposition process employing SiH_4_ as a silicon source [24,25]. Based on the findings of previous studies [26,27], the length (3 to 4 μm) and the diameter (60 to 100 nm) of the nanowires were set to optimum cell interfacing conditions.

### Cell culture and fixation

An autoclave and ethanol were used to sterilize the substrates, and the substrate surfaces were chemically modified by a poly-L-lysine (PLL) coating for cell adhesion. Primary hippocampal neurons, or GH3 cells, were cultured on the substrates in a 24-well plate at 37°C in a 5% CO_2_ incubator. They were observed using a scanning electron microscope (SEM) and treated via a critical point drying technique after glutaraldehyde (for fixation) and osmium tetroxide (for contrast enhancement) treatments.

## Results and discussion

Si nanowires were chosen as building blocks to probe neural cells because crucial factors for intracellular interfacing, such as their diameter, length, etc., can be easily tuned. Moreover, our previous study indicated that Si nanowires are bio-compatible to excitable cells (hippocampal neurons) and are thus safe for interfacing [26].

**Figure 1:**
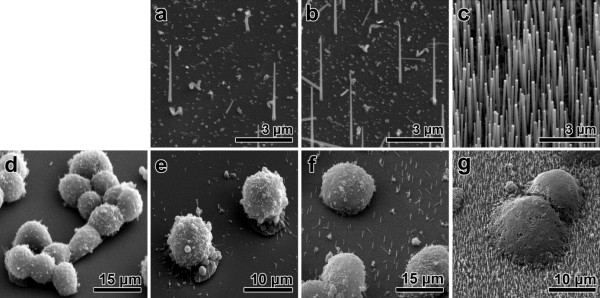
**Scanning electron microscope images of Si nanowires and GH3 cells. (a,b,c)** Typical SEM images of Si nanowires grown on a Si substrate with various wire densities (**(a)** 2.5 × 10^4^ mm^−2^, **(b)** 1.5 × 10^5^ mm^−2^, **(c)** 1.5 × 10^6^ mm^−2^). **(d,e,f)** SEM images of GH3 cells cultured on plane Si and nanowire-grown substrates shown in **(a)**, **(b)**, and **(c). (g)** SEM images of GH3 cells cultured on Si nanowire-grown substrates with high population density.

It is known that the cell process is critically affected by the surface that the cells come into contact with [28-30]. In our study, the nanowire population density, diameter, and length were investigated because they determine the surface structure of the substrate. Figure 1a,b,c shows nanowires grown on substrates with densities of Figure 1a 2.5 × 10^4^ mm^−2^, Figure 1b 1.5 × 10^5^ mm^−2^, and Figure 1c 1.5 × 10^6^ mm^−2^. Figure 1d,e,f,g shows SEM images of GH3 cells cultured on bare silicon substrate and the three substrates noted above for 72 h. In the bare silicon substrate, as shown in Figure 1d, GH3 cells were attached loosely to the silicon surface and grew close to other cells. Figure 1e,f,g shows that the cell body appeared to be widely stretched and attached tightly as the population density of nanowires increases. In the case of the substrate with the low population density of nanowires, most of the cells grew normally and displayed a morphology equivalent in quality to that grown on the bare silicon substrate without regard to nanowire interfacing. In the case of the interfacing with the high population density of nanowires, we observed some cells with a holey membrane as shown in Figure 1g, indicating a loss of their functions. This means that GH3 cells failed to withstand wiring damage.

To verify how nanowire interfacing affects the cell viability, an MTT assay, a technique widely used to measure cell viability, was performed under the same conditions. Additional file 1: Figure S2 shows that the activity of the GH3 cell interfaced with a certain nanowire density and culture time is higher than that cultured on the bare silicon substrate. It also shows that too many interfaces with nanowires can have an adverse effect on the cell viability.

We investigated the effect of the population density of the nanowires on the growth of primary hippocampal neurons. At low nanowire density, as shown in Additional file 1: Figure S3d of supplementary data, hippocampal neurons displayed a normal morphology equivalent in quality to grown on the flat substrate. Their processes are well-developed in number and size. The figures also show that the nanowires penetrated the neural body. Under this intracellular interfacing, the entire cell membrane is complete and undamaged, retaining a structural functionality despite the distinct penetration of nanowires from the bottom to the top of the neuron cells. In the case of moderate density, hippocampal neurons failed to withstand wiring damage, as shown in Additional file 1: Figure S3e of supplementary data. The figure shows that many cells were destroyed, losing their original shape. The cell debris was tangled with nanowires in many locations. This indicates that the primary cell had grown and developed for some time after cell seeding. On the substrate with the highest nanowire density, hippocampal neurons showed no growth and remained embryonic in shape (Additional file 1: Figure S3f of supplementary data). This reveals that cells have specific tolerance toward the amount of nanowire penetration. GH3 cells are more active and thus are not as sensitive to the density of the nanowires as hippocampal neuron cells.

Previous studies indicate that probing cells using electronic devices are highly sensitive to the types of interfaces, as the most critical point in signal transfer from the cell to the device is the interface between these two domains [31-34]. In particular, the interface should have no cleft in order to allow signal transfer. The intracellular interfaces between nanowires and cells have not been investigated, and thus, these were examined in this study. Additional file 1: Figure S4a of supplementary data shows a schematic drawing of the cross-sectioning process. The intracellular coupled interfaces were cross-sectioned parallel to the longitudinal direction of the nanowires using a high-resolution Cross Beam focused ion beam field emitted SEM (FIB-FESEM). The sidewall was polished with a low ion current and imaged by SEM in an *in situ* mode. Additional file 1: Figure S4b of supplementary data shows a SEM image of the neuron-nanowire interface from the cross-section parallel to the longitudinal direction of the nanowires. The entire cross-sectional interfacial structure was well preserved, and distinct shrunken artifacts were not found. The nanowire penetrated the neuron membrane, which is attached tightly to the nanowires. These outcomes indicate that Si nanowires with diameters of <100 nm, lengths of several micrometers, and approximate densities of 2.5 × 10^4^ mm^−2^ can achieve intracellular interfacing with excitable cells in a living state with tight interfaces without any cleft. This result implies that they may be suitable for probing excitable cells in an intracellular mode. Meanwhile, CNT array properties, i.e., conductivity, diameter, and length, are difficult to control for them to suit the intracellular interfacing or single cell signaling experiments. Patch clamp is conventional equipment for intracellular single cell signaling. The probe size of patch clamp is micro-scale, and the cell membrane should be broken for the probe and cell interfacing. Therefore, patch clamp is not suitable for *in vivo* experiment and neuronal interfaces between neuron.

**Figure 2:**
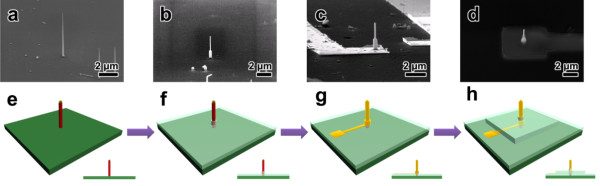
**SEM images and a schematic bird's eye view of the build-up procedure of the vertical nanowire probe electrode. (a,b,c,d)** SEM images of the build-up procedure of the vertical nanowire probe electrode (**(a)** selected vertical nanowire, **(b)** bottom passivation layer preventing electrical leakage, **(c)** Pt deposition for electrode formation, **(d)** top passivation layer for intracellular recording, scale bar is 2 μm]. **(e,f,g,h)** A schematic bird's eye view of the build-up procedure of the vertical nanowire probe electrode (inset: cross-sectional view).

Nanowire probes were fabricated based on the results. Si nanowires with optimum conditions (diameter of 60 nm, length of 3 to 4 μm, density of 2.5 × 10^4^ mm^−2^) were grown vertically on a highly resistive intrinsic Si substrate (shown in Figures 1a and 2a). These Si nanowires are single crystalline, and the growth direction of nanowire is the (111) axes that are perpendicular to the (111) planes of face-centered cubic structure (See Additional file 1: Figure S1 of supplementary data). A working field of 120 μm × 120 μm was defined to make an alignment mark on the substrate for photolithography and sputter deposition. A photoresistor (PR) was then coated on the substrate with polymethylglutarimide (PMGI) and AZ 5214E by spin-coating and baking, respectively. The substrate was then sonicated in distilled water to remove dispensable nanowires, and a vertical Si nanowire was selected with reference to a pre-defined coordinate system, using an FESEM. An initial SiO_2_ dielectric layer approximately 700-nm thick was deposited by high-density plasma chemical vapor deposition (HDP CVD), and the nanowires were exposed by a wet etching process using an ammonium fluoride mixture (shown in Figure 2b). This SiO_2_ dielectric layer prevents the flow of leakage current from the nanowire probes to the substrate, which appears to be crucial to achieve very tiny signals from each probe.

Cr/Pt electrodes, which are connected with an external circuit, were then defined using photolithography and a sputtering process. A Pt layer that acts as an active electrode for signaling was subsequently defined for the individual nanowires by e-beam lithography and a sputtering process (shown in Figure 2c). This step was necessary because Si nanowires have a native SiO_2_ layer with thickness of 2 nm. This layer would build a very high potential barrier for signal transfer between the cell and nanowire probe. A second SiO_2_ layer was deposited via an HDP CVD process in order to electrically isolate the entire electrode from buffer solution and protect the electrodes from the chemical reaction in the wet cell culturing medium. Finally, the second passivation layer on the top part of nanowire probe was etched selectively by blocking the rest of the probe, which was wrapped with polymethyl methacrylate. This anisotropic wet etching method makes the nanowire probe have a suitable structure for intracellular recording (shown in Figure 2d).

The electrical properties of the nanowire probes were characterized by measuring the cyclic voltammograms (CV) (Additional file 1: Figure S5 of supplementary data). CVs were measured with a Pt counter electrode and Ag/AgCl was used as a reference electrode. No decrease of current after a small peak was observed in our nanoelectrode. Such a behavior is common in nanosize electrodes since analytes diffuse according to hemispherical diffusion in electrodes, which leads to a higher mass transport per unit electrode surface. The sigmoidal voltammograms, which show limiting current, are characteristic of radial diffusion to cylindrical ultramicroelectrodes. Assuming that the electrode is a cylindrical shape, the limiting plateau currents can be determined according to the following equation [35].

(1) $$I=4\pi \mathrm{nFDCl}\frac{1}{\ln\;\left[\frac{4\left(\mathrm{Dt}\right)}{r_{0}^{2}}\right]}.$$

Here, *n* is the number of electrons transferred during the electrochemical process, *F* is Faraday's constant, *D* and *C* are the diffusion coefficient and concentration of the electroactive species respectively, *l* and *r* are the length and radii of nanoelectrode, respectively, and *t* is time scale of the CV experiment, which is represented by RT/Fv. The experimental limiting current value at our nanoelectrode is 4.5 nA, which is similar to the theoretical limiting current value (4.21 nA/μm).

**Figure 3:**
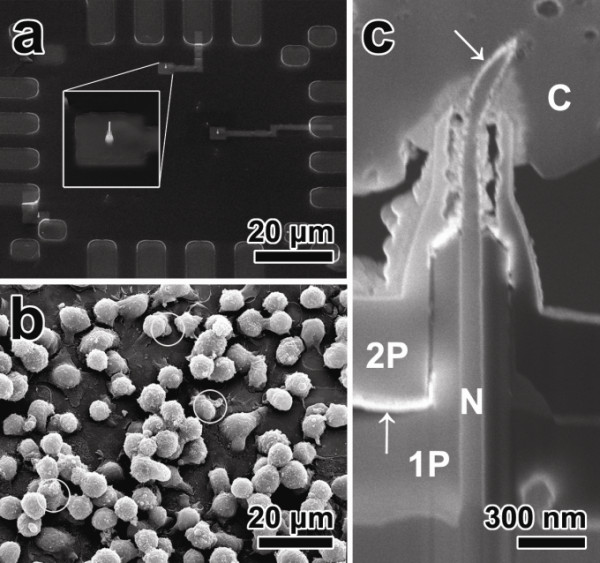
**SEM images of the fabricated device's center, GH3 cell, and cross-sectional nanowire probe-cell interface. (a)** An SEM image of the center part of the fabricated device (inset: magnification of vertical nanowire probe). **(b)** An SEM image of a GH3 cell cultured on the device (white circle: the position of vertical nanowire probe). **(c)** An SEM image of a cross-sectional nanowire probe-cell interface (N: nanowires, C: GH3 cell, 1P: bottom passivation layer, 2P: top passivation layer, white arrows: Pt layer).

The probing of neural activity was carried out using a rat clonal GH3 pituitary cell line, which has a spontaneous action potential that is known to be stimulated by a thyrotropin releasing hormone [36]. As such, it is ideal to test the feasibility of the nanowire probe for measuring neural activity without external stimulation to induce an action potential. Figure 3a is an SEM image of the vertical nanowire probe device before the culturing of the GH3 cells. Culturing was carried out with GH3 cells 2 days after cell plating. The standard bath solution consisted of 140 mM NaCl, 5 mM KCl, 2 mM CaCl_2_, 1 mM MgCl_2_, 10 mM 4-(2-hydroxyethyl)-1-piperazineethanesulfonic acid (HEPES), and 10 mM glucose was applied continuously into the culturing bath through a gravity-fed perfusion system during recording. Measurements were carried out at 25°C. Figure 3b is an SEM image of GH3 cultured in the same location as that shown in Figure 3a by seeding the cells of passage 10. The white circles in Figure 3b indicate the sites where the vertical nanowire probes are positioned. The image clearly shows that the nanowire probes are covered with GH3 cells. The individual probing electrode was connected to the input of a buffer. It was then connected to the bridge circuit of an electrophysiology amplifier (Axoclamp-900A, Axon Instruments, Jakarta Selatan, Indonesia), which was connected to a digitizer (Digidata1440, Molecular Devices, Sunnyvale, CA, USA) attached to a personal computer running pClamp10. The bath was grounded with a Ag/AgCl electrode immersed in the bath solution, and the voltage signals were monitored in current-clamp mode and filtered at 3 kHz.

**Figure 4:**
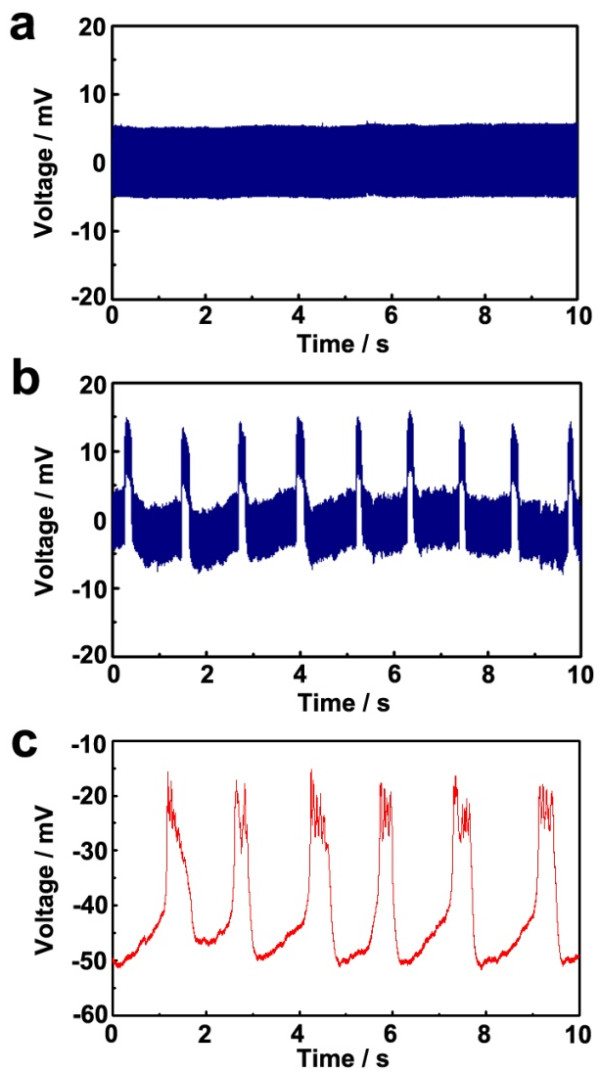
**Graphs of the voltage change and the signal of GH3 cells. (a,b)** Graphs of the voltage change via vertical nanowire probe device in the current-clamp mode (**(a)** no cell, **(b)** GH3 cell). **(c)** The signal of GH3 cells acquired from the conventional patch clamp system at the current-clamp mode.

Figure 4a shows the signal without GH3 cells, revealing a baseline signal with no events. The background noise is roughly at a level of ±5 mV and may be due to relatively high resistance of the nano-sized probe. Figure 4b shows the signal from a vertical nanowire probe with GH3 cells, presenting a series of spontaneous positive deflections. These peaks, which arise from a spontaneous action potential of GH3 cells, rapidly reached a steady state with average peak amplitude of approximately 10 mV, duration of approximately 140 ms, and period of 0.9 Hz. In the course of the signal detection, we could ignore the interference signals from near GH3 cells, because the interference signals of neighboring GH3 cells are the extracellular signal of micro-voltage level [37-39]. Also, because the nanowire probe is located in the GH3 cell and the probe is packed with the cell membrane, the external signals of the neighboring cells are hard to the interference. The duration and period of the peak of the signal are similar to that of the patch clamp signal in GH3 cells (shown in Figure 4c). The amplitude of the signal is smaller than that from the patch clamp, possibly due to the resistance of the vertical probe device. According to the equivalent circuit (Additional file 1: Figure S6 of supplementary data), the cell membrane potential is distributed between the electrode and differential amplifier resistances. Since a voltage drop occurred in the vertical nanowire probe device around the cell/nanowire probe interfaces with relatively high resistances compared to that of the head-stage probe, the amplitude is expected to be smaller than that from the patch clamp.

After signal recording, the coupled vertical nanowire probe-cell was investigated to clarify whether the nanowire probe penetrates the GH3 cell, which is essential for intracellular signaling. Figure 3c shows the cross-section of the coupled interface prepared by a high-resolution Cross Beam FIB-FESEM. Two passivation layers that coated the nanowires and a Pt layer for signal collection at the tip of the nanowires can be clearly seen in the cross-section. It is noted that the nanowire probe pierced through the cellular membrane in a bent shape, possibly due to compression by the weight of the cells. A robust passivation layer also acts as a buttress, which supports a nanowire against the cell. Figure 3c also shows that the membranes of the cells perforated by the vertical nanowire probe adhere closely to the top passivation layer without any voids. This tight coupling of the membrane and the SiO_2_ layer prevent the cytoplasm of the GH3 cell from mixing with the culture medium and the standard bath solution. By thus isolating the cells physically, it is possible to record the electrical activity inside of the cell in an intercellular mode.

## Conclusion

We demonstrated a vertical nanowire probe can be used as a tool for intracellular probing of the electrical activity of single cells. The results indicate that interfacing of vertical grown nanowires with neuronal cells (i.e., intercellular penetration), which is essential to probe living cells in an intracellular mode, can be successfully achieved by controlling the diameter, length, and density of the nanowires. It has been demonstrated that the device structure, which consisted of passivation layers and signal collector layers, is mechanically robust and can overcome the mechanical resistance from the cells and is also electrically workable for probing the action potential. It is also shown that intracellular signaling is possible, because the nanowire probe is interposed in the GH3 cell and the cell membrane is tightly attached to the passivation layer. There have been previous studies involving vertical nanowire array electronic devices [40-42] indicating the feasibility of producing vertical nanowire probes on a large scale. The outcomes of this study can be easily extended to the signaling of neural networks such as cultured primary neurons or brain slices, where it is necessary to measure long-term cellular activity in a large working area [43,44].

## Competing interests

The authors declare that they have no competing interests.

## Authors' contributions

KY, IS, SE, and DW carried out the device fabrication, cell culturing, and signalling. JJ, HR, and SH participated in the design of the study. JP carried our TEM works. HJ conceived of the study and participated in its design and coordination. All authors read and approved the final manuscript.

## Supplementary Material

Additional file 1: Figure S1 TEM images of the synthesized Si nanowires. (a) Low magnitude TEM image of the Si nanowire. The diameter of Si nanowire is approximately 60 nm. (b) High-Resolution TEM image of the Si nanowire. The inset of Additional file 1: Figure S1b is a SAED pattern of the Si nanowire. The SAED pattern indicates the Si nanowire have a single crystalline nature and the [111] growth direction. **Figure S2.** MTT assay result of GH_3_ cells interfaced with nanowire-grown substrates in various densities (PS: plane substrate, LDSN, MDSN and HDSN: nanowire-grown substrate shown in Figure 1a, 1b and 1c). **Figure S3.** SEM images of primary hippocampal neurons cultured on nanowire-grown substrates in order of Figure 1a, 1b and 1c. A white circle in d indicates penetrated nanowire from bottom to top membrane of neuron. **Figure S4.** (a) A schematic drawing for observation of cell/nanowire interface. Dotted line represents a sectioning direction of FIB. Square part is the area we observed by SEM (b) SEM images of primary hippocampal neurons-nanowire interface (N: nanowire, P: platinum layer for the protection of upper part of cell, C: cell soma). Arrow indicates cell membrane, which is covered by gold layer for a first SEM observation. **Figure S5.** Cyclic voltammogram of individual nanoelectrode in 0.1 M K_3_Fe(CN)_6_. Ag/AgCl electrode was served as the reference electrode and a platinum wire was served as the auxiliary electrode. The scan rate was 10 mV/s. **Figure S6.** Equivalent circuit of our measurement system (C_m_: cell membrane capacitance, E_m_: cell membrane potential, R_m_: cell membrane resistance, R_leak_: junction leakage resistance, R_e_: electrode resistance, C_e_: electrode capacitance). Click here for file
