# Supplementary material for: Vertical nanowire probes for intracellular signaling of living cells
Source: Nanoscale Res Lett. 2014 Feb 3;9(1):56. doi: 10.1186/1556-276X-9-56 (PMC3917366; doi:10.1186/1556-276X-9-56)
Supplement: Additional file 1: Figure S1 — TEM images of the synthesized Si nanowires. (a) Low magnitude TEM image of the Si nanowire. The diameter of Si nanowire is approximately 60 nm. (b) High-Resolution TEM image of the Si nanowire. The inset of Additional file 1: Figure S1b is a SAED pattern of the Si nanowire. The SAED pattern indicates the Si nanowire have a single crystalline nature and the [111] growth direction. Figure S2. MTT assay result of GH3 cells interfaced with nanowire-grown substrates in various densities (PS: plane substrate, LDSN, MDSN and HDSN: nanowire-grown substrate shown in Figure 1a, 1b and 1c). Figure S3. SEM images of primary hippocampal neurons cultured on nanowire-grown substrates in order of Figure 1a, 1b and 1c. A white circle in d indicates penetrated nanowire from bottom to top membrane of neuron. Figure S4. (a) A schematic drawing for observation of cell/nanowire interface. Dotted line represents a sectioning direction of FIB. Square part is the area we observed by SEM (b) SEM images of primary hippocampal neurons-nanowire interface (N: nanowire, P: platinum layer for the protection of upper part of cell, C: cell soma). Arrow indicates cell membrane, which is covered by gold layer for a first SEM observation. Figure S5. Cyclic voltammogram of individual nanoelectrode in 0.1 M K3Fe(CN)6. Ag/AgCl electrode was served as the reference electrode and a platinum wire was served as the auxiliary electrode. The scan rate was 10 mV/s. Figure S6. Equivalent circuit of our measurement system (Cm: cell membrane capacitance, Em: cell membrane potential, Rm: cell membrane resistance, Rleak: junction leakage resistance, Re: electrode resistance, Ce: electrode capacitance). [file 1556-276X-9-56-S1.docx]

Electronic supplementary data for Nanoscale Research Letters

**Additional File 1**

**Vertical Nanowire Probes for Intracellular Signaling of Living Cells**

Ki-Young Lee^1, 2†^
Email: redcrono@gmail.com

Ilsoo Kim^1†^
Email: nicepolo@yonsei.ac.kr

So-Eun Kim^1^
Email: k.ssogood@gmail.com

Du-Won Jeong^3^
Email: dowone@jbnu.ac.kr

Ju-Jin Kim^3^
Email: jujinkim@jbnu.ac.kr

Hyewhon Rhim^4^
Email: hrhim@kist.re.kr

Jae-Pyeong Ahn^5^
Email: jpahn@kist.re.kr

Seung-Han Park^6^
Email: shpark@yonsei.ac.kr

Heon-Jin Choi^1,*^
* Corresponding author
Email: hjc@yonsei.ac.kr

†Equal contributors

^1^ Department of Materials Science and Engineering, Yonsei University, Seoul 120-749, Republic of Korea

^2^ Spin Convergence Research Center, Korea Institute of Science and Technology, Seoul 139-791, Republic of Korea

^3^ Department of Physics, Chonbuk National University, Jeonju 561-756, Republic of Korea

^4^ Center for Chemoinformatics Research Center, Korea Institute of Science and Technology, Seoul 139-791, Republic of Korea

^5^ Advanced Analysis Center, Korea Institute of Science and Technology, Seoul 139-791, Republic of Korea

^6^ Department of Physics, Yonsei University, Seoul 120-749, Republic of Korea

**
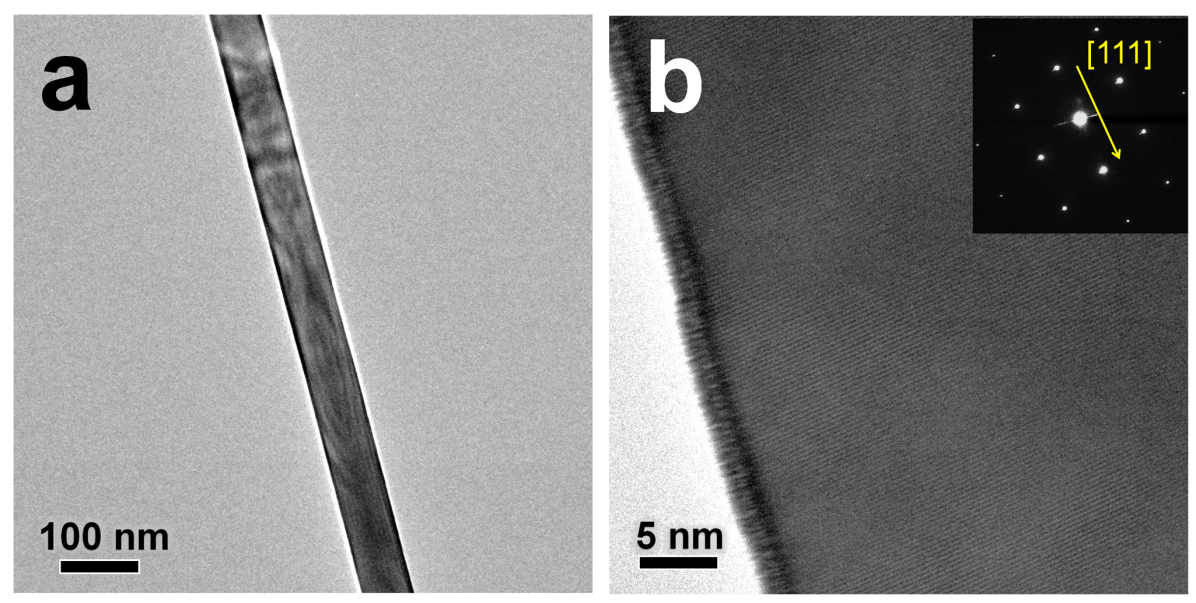
**

**Figure S1.** TEM images of the synthesized Si nanowires. (a) Low magnitude TEM image of the Si nanowire. The diameter of Si nanowire is approximately 60 nm. (b) High-Resolution TEM image of the Si nanowire. The inset of Figure S1b is a SAED pattern of the Si nanowire. The SAED pattern indicates the Si nanowire have a single crystalline nature and the [111] growth direction.

.


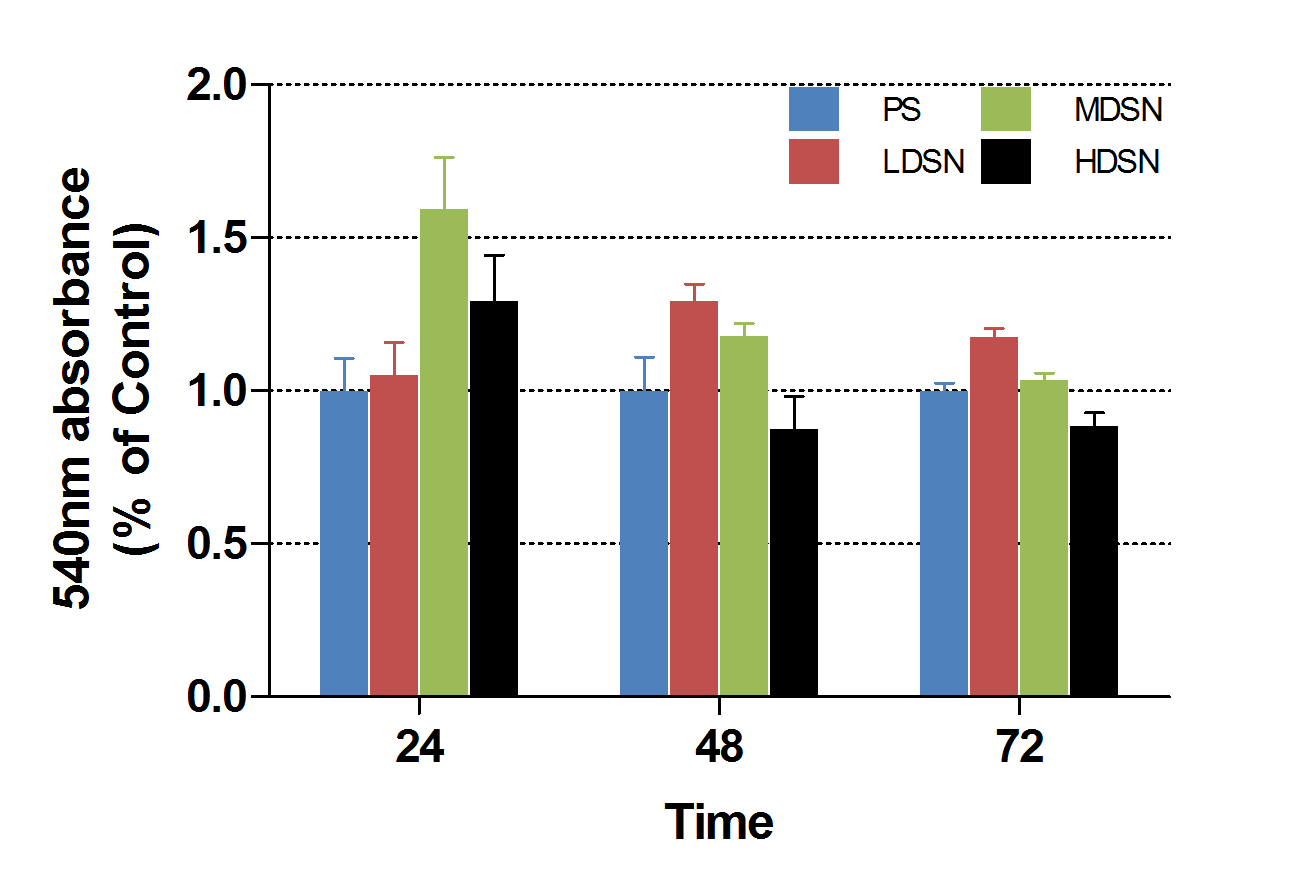
**Figure S2.** MTT assay result of GH_3_ cells interfaced with nanowire-grown substrates in various densities (PS: plane substrate, LDSN, MDSN and HDSN: nanowire-grown substrate shown in figure 1a, 1b and 1c).


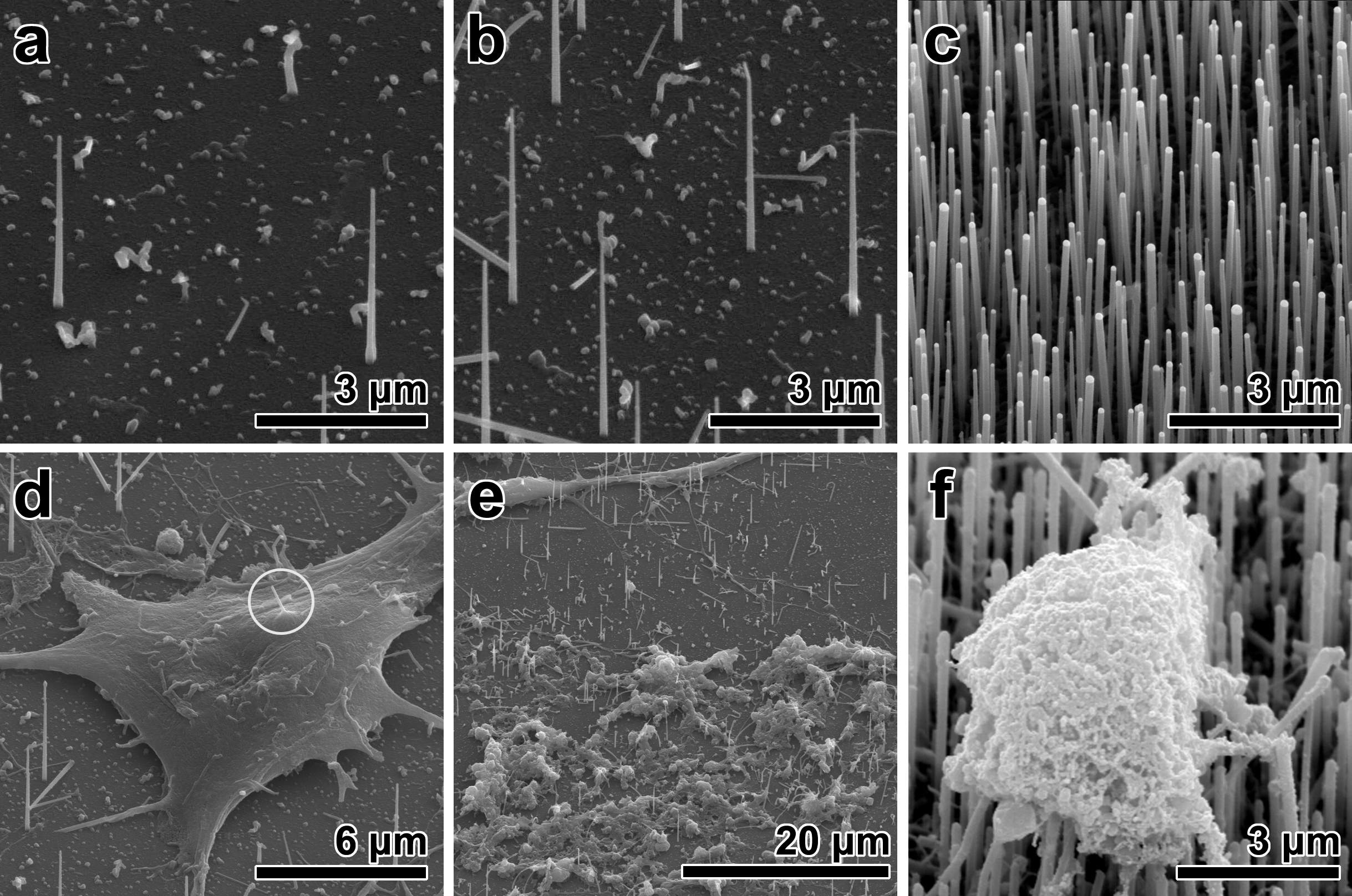


**Figure S3.** SEM images of primary hippocampal neurons cultured on nanowire-grown substrates in order of figure 1a, 1b and 1c. A white circle in d indicates penetrated nanowire from bottom to top membrane of neuron.


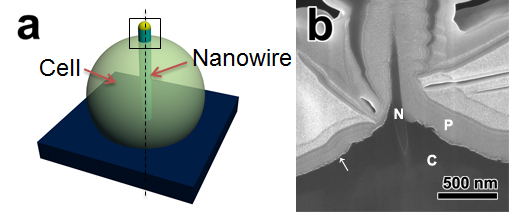


**Figure S4.** (a) A schematic drawing for observation of cell-nanowire interface. Dotted line represents a sectioning direction of FIB. Square part is the area we observed by SEM (b) SEM images of primary hippocampal neurons-nanowire interface (N: nanowire, P: platinium layer for the protection of upper part of cell, C: cell soma). Arrow indicates cell membrane, which is covered by gold layer for a first SEM observation.


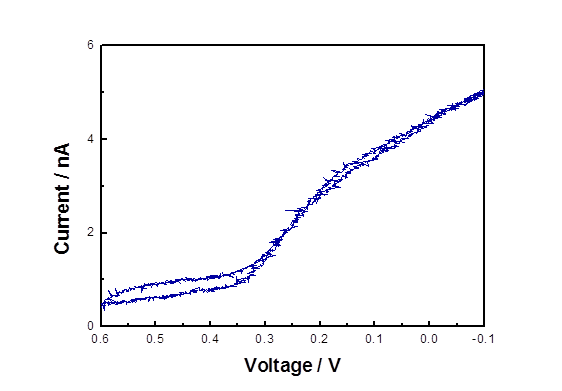
**Figure S5.** Cyclic voltamogram of individual nanoelectrode in 0.1 M K_3_Fe(CN)_6_. Ag/AgCl electrode was served as the reference electrode and a platinum wire was served as the auxiliary electrode. The scan rate was 10 mV/s.


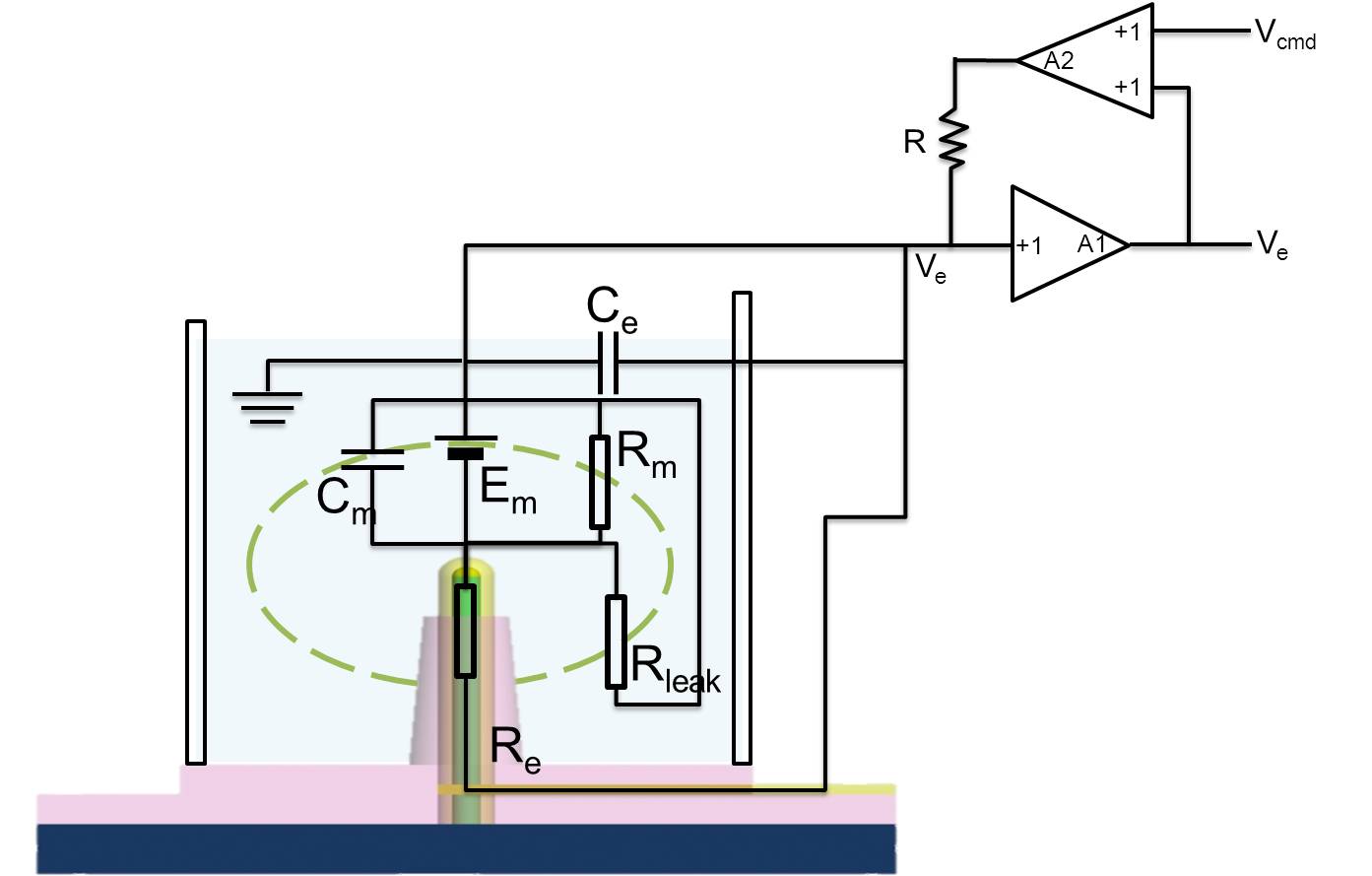


**Figure S6.** Equivalent circuit of our measurement system (C_m_: cell membrane capacitance, E_m_: cell membrane potential, R_m_: cell membrane resistance, R_leak_: junction leakage resistance, R_e_: electrode resistance, C_e_: electrode capacitance).
